# An Academic Viewpoint (2025) on the Integration of Generative Artificial Intelligence in Medical Education: Transforming Learning and Practices

**DOI:** 10.7759/cureus.81145

**Published:** 2025-03-25

**Authors:** Mohammad Almansour, Mona Soliman, Raniah Aldekhyyel, Samar Binkheder, Mohamad-Hani Temsah, Khalid H Malki

**Affiliations:** 1 Department of Medical Education, College of Medicine, King Saud University, Riyadh, SAU; 2 Department of Pediatrics, College of Medicine, King Saud University, Riyadh, SAU; 3 Pediatric Intensive Care Unit, Department of Pediatrics, King Saud University Medical City, Riyadh, SAU; 4 Research Chair of Voice, Swallowing, and Communication Disorders, Department of Otolaryngology-Head and Neck Surgery, College of Medicine, King Saud University, Riyadh, SAU

**Keywords:** adaptive learning, ai-driven assessment, ai in healthcare, algorithmic bias, clinical training, data privacy in ai, ethical ai implementation, generative artificial intelligence, medical education, personalized learning

## Abstract

Generative artificial intelligence (GAI) has introduced a new era of medical education by offering innovative solutions to critical challenges in teaching, assessment, and clinical training. This expanded review explores the current and potential applications of GAI across multiple domains, including personalized tutoring, enhanced academic administrative efficiency, and improved preparedness for daily learning interactions. Utilizing a narrative review methodology combined with expert analysis, this study involved a structured literature search in January 2025 across PubMed, Scopus, and Google Scholar, followed by iterative brainstorming sessions and expert evaluations to assess the feasibility and impact of various GAI applications. Six domain experts then appraised the feasibility and impact of GAI technologies across educational settings, resulting in 10 identified domains of application: Quality and Administration, Curriculum Development, Teaching and Learning, Assessment and Evaluation, Clinical Training, Academic Guidance, Student Research, Student Affairs, Internship Management, and Student Activities. Our findings highlight how GAI supports personalized learning - through adaptive tutoring and automated performance dashboards - while optimizing administrative tasks such as course registration and policy oversight. In addition, immersive simulations and virtual patient encounters reinforce clinical decision-making and practical skills. GAI-driven tools also streamline research processes via automated literature reviews and proposal refinement, ultimately fostering greater efficiency across academic environments. Despite these opportunities, ethical considerations remain a priority. Issues pertaining to data privacy, algorithmic bias, and equitable access must be addressed through robust regulatory frameworks and institution-wide policies. Overall, by embracing targeted, ethically guided implementations, GAI has the evolving potential to enhance educational quality, improve operational effectiveness, and equip future healthcare professionals with the adaptive skills needed in a patient-centered clinical landscape.

## Introduction and background

The rapid evolution of digital technology and artificial intelligence (AI) has spurred significant changes in medical education, prompting educators to seek innovative methods to enhance learning outcomes. Traditional education models, often criticized for their limited scalability and static content delivery, are increasingly being challenged by generative artificial intelligence (GAI) tools. Unlike traditional AI, which primarily relies on rule-based systems or predictive analytics, GAI is designed to create new content, adapt dynamically to user input, and generate human-like responses, making GAI particularly relevant in medical education. These tools, including personalized learning platforms, automated assessment systems, and virtual clinical training modules, offer a transformative approach by tailoring educational experiences to individual learners’ profiles and needs [1]. As the healthcare landscape becomes more complex, integrating these technologies not only supports a deeper understanding of intricate subjects but also fosters an environment where learning is both adaptive and responsive to the dynamic demands of modern clinical practice.

Several recent studies have highlighted the potential of GAI to revolutionize various facets of medical education. For instance, Strielkowski et al. demonstrated how AI-driven adaptive learning systems can catalyze sustainable educational transformation by aligning content with student interests and learning styles [1]. In parallel, Hamilton’s work on healthcare simulation illustrates the pivotal role of virtual clinical training tools in enabling students to practice decision-making and diagnostic skills within realistic, simulated patient scenarios [2]. Zheng and Mavis further reinforced the value of case-based learning approaches in bridging the gap between theoretical knowledge and practical application [3] while Maluleke et al. explored the benefits of cross-disciplinary mathematical modeling in enhancing clinical pharmacology education [4].

Beyond the immediate advantages in personalized learning and clinical training, the integration of GAI in medical education holds promise for alleviating faculty workload through automated grading and administrative assistance [5]. This dual benefit of improving both the quality of education and operational efficiency underscores the importance of adopting innovative, technology-driven solutions in academic settings. However, these advancements are not without challenges. Ethical considerations, such as the potential for algorithmic bias, concerns over data privacy, and the need for transparency in AI decision-making, must be carefully managed to ensure that the deployment of these systems is both responsible and equitable [5,6].

This narrative review aims to provide an updated and comprehensive overview of current GAI applications in medical education and to explore their potential to transform teaching, learning, and clinical practice. By synthesizing findings from diverse studies and expert analyses, the review seeks to offer an academic viewpoint that not only highlights the transformative potential of these technologies but also addresses the critical challenges that must be overcome to fully realize their benefits.

## Review

Methods

This review adopted a narrative review methodology combined with expert analysis to identify and evaluate GAI applications used in medical education. The process began with a literature search conducted in January 2025 across PubMed, Scopus, and Google Scholar using the terms “AI in medical education,” “adaptive learning,” and “clinical simulations.” Following this, three iterative brainstorming sessions were held on January 30, 2025, where the authors compiled a comprehensive list of GAI applications.

**Figure 1:**
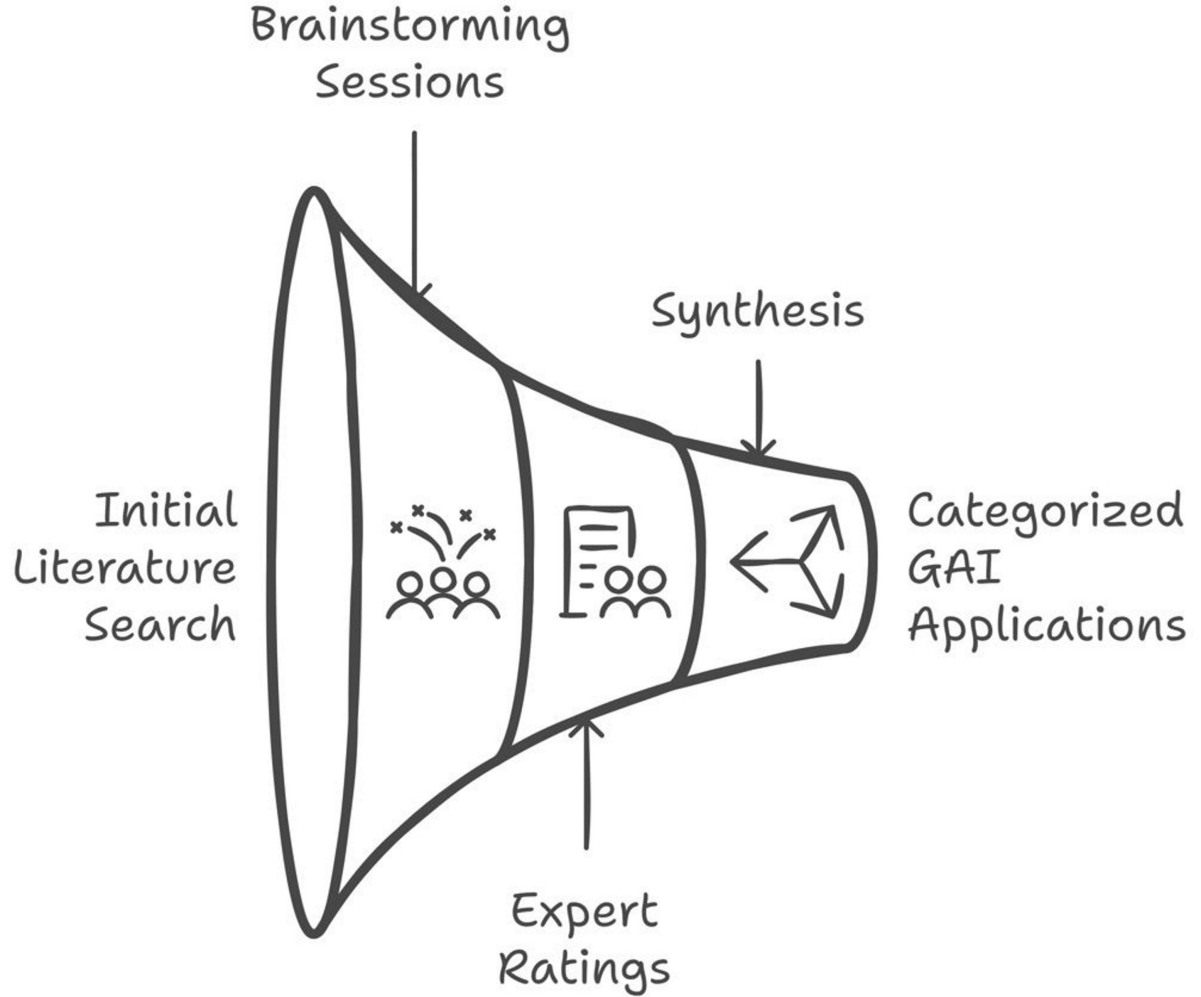
Review methodology combined with expert analysis for generative artificial intelligence (GAI) applications used in medical education Image credit: Mohamad-Hani Temsah

To assess the feasibility and priority of each application, six domain experts provided ratings using a three-point scale ranging from 1 to 3. Finally, the findings were synthesized and categorized into 10 domains based on their relevance and potential impact. Figure 1 illustrates this process in detail.

Results

Key Domains for AI Application in Medical Education

1.Quality and Administration

AI-driven policy guidance: Custom GAI systems that leverage natural language processing and predictive algorithms can provide accurate, instant responses to standard questions from students, update administrative processes like policy changes and support, and create a record of frequently asked questions. For example, GAI tools could help individuals select classes to meet complex curriculum requirements or inform them of grading policies with real-time data integration from institutional databases [7,8].

Curriculum benchmarking: AI tools can be used to examine a curriculum’s compliance with international standards by systematically analyzing learning objectives, course structures, and outcomes [9]. These tools may assess institutional curricula’s alignment with global benchmarks of accreditation bodies or top-tier universities. This will help institutions identify the strengths and weaknesses of various curricula and develop appropriate strategies to compete for student enrollment. Moreover, these tools could be used to identify trends in medical education, such as personalized medicine and digital health technologies, ensuring that the programs are still innovative and technology-proof in the future.

Automated performance dashboards: AI-based systems can be created that can track the performance of faculty and students in real-time [10]. These dashboards can compile data from many sources, such as test scores, attendance sheets, and student feedback forms, to develop a set of reports that can be provided to the students. This tool can help teachers identify students who are struggling or need extra help. Furthermore, the students can receive specific recommendations based on their performance. The dashboards can also indicate changes over time. Thus, college heads can tailor curricula and teaching methods to fit the organizational objectives.

2. Curriculum Development and Design

Gap analysis: Cutting-edge AI technology can be used to identify areas of the curriculum that require improvement. AI programs will compare a facility’s educational outcomes or report cards to global health trends and accreditation standards as well as the student competency framework [11]. These computer-based tools can scrutinize a subject’s courses, assessment results, and students’ opinions to reveal areas that need improvement. For example, AI will identify less common topics within a course, such as digital health and medical technologies, to create a knowledge spectrum that is comprehensive and globally relevant.

AI-driven learning objectives: AI leverages its technologies to set and achieve goals in specific fields by molecularly screening the course content and identifying gaps [12]. These AI tools can also propose alterations in the course content or teaching methods to encourage the global incorporation of high teaching and learning standards in medical education. Furthermore, AI-based systems can help foster the integration of competency frameworks and illustrate the learning outcomes visually through performance dashboards that automatically help educators track students’ progress and customize their teaching. These systems also provide teachers with multifunctional tools to monitor student improvement, refine learning outcomes, and provide a complete educational experience.

3. Teaching and Learning

Dynamic multimedia creation: AI tools can create engaging animated educational material, such as video lectures, graphics, and quizzes, as well as helping in creating educational Microsoft PowerPoint presentations [13]. They present materials that allow teachers to provide specifically targeted content tailored to each student. For example, AI will organize explanatory videos, quizzes, and animations related to several objectives. This program can also be linked to a live statistical data display so that lecturers can instantly evaluate it.

Virtual patient encounters: AI-powered platforms allow students to develop communication and diagnostic skills needed for patient interactions through simulations [14], enabled by enhancing technology for virtual, scenario-based training. Through these platforms, students can engage with virtual patients exhibiting various symptoms and conditions, allowing them to learn in a clinical setting. In this way, the platforms contribute to developing clinical reasoning, empathy, and decision-making skills. Cutting-edge AI algorithms can analyze the student’s mental state from their input and provide automated patient responses. These abilities make the environment accurate and dynamic. In addition to their instant feedback and analytics, these tools enable students to see the mistakes they have made. Thus, they improve students’ confidence in dealing with complex clinical situations in their practice.

Advanced feedback mechanisms: The systems with the most up-to-date AI technologies can provide feedback about assessments (e.g., to students and employees). These are known as advanced feedback mechanisms, and they focus on enabling students to perform their best [15]. Smart deployment and the use of AI and machine learning technologies are the cornerstones of such systems. They analyze all users’ responses separately, recommend a specific range of enhancement actions, and provide users with evolutions of their performance over time in elaborate reports. Change occurs when students take charge of their learning, make informed decisions, and practice focused analysis.

4. Assessment and Evaluation

Interactive question banks: Custom AI systems create various question types, including MCQs, case-based assessments, and adaptive testing scenarios [8,16]. AI-driven question banks can measure students’ skills and create personalized question sets from the data, targeting their weak areas with increasingly challenging questions to improve their understanding. Moreover, the systems can simulate scenarios in which students must make decisions. In this way, students’ clinical reasoning skills and adaptability are tested.

Simulated OSCEs: Next-generation AI technologies and devices can be applied for speech perception assessment and interpersonal communication (both face-to-face and virtual) during OSCE exams [17]. These tools not only determine the tone, clarity, and confidence of a student’s response but also assess non-verbal communication, including body language and facial expressions. AI algorithms can provide comments with detailed reasoning, which will allow students to identify their strengths and areas for improvement. These programs also allow students to explore multiple languages with complete translations, broadening their clinical horizons.

Plagiarism detection: AI tools assess the uniqueness of textual materials by applying algorithms to compare texts with databases, online libraries, and schools [18]. These tools can pinpoint potentially plagiarized sections in documents, present detailed similarity reports, and make suggestions to ensure the originality of the documents. Their utilization as part of learning management systems (LMSs) challenges learners and ensures academic integrity and proper citation practices, improving the students’ education.

5. Clinical Training

Rare case simulations: AI can create rare clinical conditions, presenting cases that challenge students because they are uncommon in clinical settings [19]. These simulations may include rare genetic disorders, atypical disease presentations, or critical care emergencies. AI programs can respond to each student’s progress with individualized cases, tailoring the entire learning process. Additionally, these simulations can be combined with virtual reality (VR) to create a realistic and highly immersive setup, allowing students to improve their diagnostic and thinking skills and prepare for real-world obstacles.

Virtual reality modules: Modern technology provides advanced VR module platforms that can re-create scenarios ranging from surgeries to diagnostic procedures, allowing students to safely and efficiently practice critical skills in a controlled environment [20]. Students can engage in chaotic scenarios created by these modules, demonstrating that the virtual environment can be as effective as real-world settings. In a simulation, learners can experience real-life clinical scenarios, fully immersing themselves in the learning process through experience rather than observation alone. VR systems can also include real-time feedback, enabling students to perfect their skills. Furthermore, these simulations can provide different levels of expertise such as beginner, intermediate, or advanced, allowing for personalized educational paths and preparing students for real-life challenges.

AI-powered image analysis: Utilizing AI, students can learn to interpret radiology and pathology images with similar accuracy to highly advanced machine learning algorithms [21]. These tools help learners detect patterns and abnormalities in radiologic or other image studies, practicing diagnostic techniques in a controlled environment during their training. Additionally, AI systems can inform learners why a diagnosis is incorrect, and they can point out the portions of an image students should focus on to make a diagnosis through real-time feedback and detailed explanations of image features. By integrating these tools into the educational program, colleges can advance students’ knowledge of image-based diagnostics, preparing them for clinical practice.

6. Academic Guidance

Custom academic pathways: AI tools can analyze students’ performance, learning styles, and factors that affect their academic goals to develop personalized learning plans tailored to individual needs and preferences [22]. AI processes each piece of information, identifying educational issues using the data it has already assessed, the records of the student’s attendance to classes, and their engagement in class meetings. In this way, the system suggests individualized study schedules, recommends additional resources, and indicates areas where students can improve. This support method ensures that students receive specific help to ensure success, enabling them to achieve their academic goals.

Targeted mentorship programs: Academic AI can help teachers provide targeted and tailored mentorship to students by providing information on individual academic performance, behavior patterns, and participation metrics analysis [23]. Unlike humans, AI can identify areas where students are doing well and those in need of improvement. Thus, they can prepare teaching materials and assignments that suit each student. In addition, AI-powered mentorship initiatives can ensure that learning is on track, allowing suitable corrections and stronger mentor-student relationships.

7. Student Research

Automating literature reviews: AI technologies can give detailed overviews of relevant material in a fraction of the time, and they can identify the most appropriate journal articles by searching extensive academic databases, extracting the main points, and sequencing them appropriately [24]. This can help academics by providing them with summaries of the main issues, ranking articles by relevance, and offering additional references. AI uses natural language processing to convert data into a comprehensible form. Thus, the user is spared from the laborious task of manual data input that otherwise makes the process inefficient and inaccurate.

Data visualization: Automated visualization tools drastically diminish the level of abstraction in statistical outputs, as they can transform complex data into clear, interactive graphs, charts, and infographic formats [25]. The software behind these AI-based technologies uses advanced data mining techniques that quickly reveal patterns, trends, and correlations, helping teachers and students understand the data more efficiently. They have various customization options that gather information for research, create presentations, and make decision-making processes more objective and well-informed.

Proposal review assistance: AI systems provide detailed, constructive feedback on research proposals by analyzing the structure, coherence, and alignment with academic standards [26]. These tools can identify methodology gaps, suggest improvements for arguments, and highlight areas needing additional references or data. Through natural language processing and machine learning algorithms, AI tools can pre-check proposals to be submitted to peer-reviewed journals, ensuring that they are high-quality and will contribute to the research field.

8. Student Affairs

AI-assisted registration: AI technologies automate elective course registration by streamlining the selection process and reducing the administrative workload of the university staff [27]. These systems can dynamically allocate available slots, prioritize selection according to student preferences and prerequisites, and ensure equitable access to high-demand courses. Moreover, AI keeps track of students participating in summer training programs and generates detailed analytics that allow institutions to monitor performance, evaluate program outcomes, and offer tailored recommendations for future academic planning.

Feedback systems: AI-powered feedback systems provide students access to their insights regarding their academic and extracurricular performance [28]. After analyzing data from assignments, exams, and activities, these systems identify strengths and areas for improvement. Advanced algorithms demonstrate value by suggesting targeted strategies, recommending resources, and tracking progress over time. In addition, these systems can provide immediate feedback, allowing students to react quickly and adapt to their learning experience in an optimal way.

GAI for mental health support: A modern trend in mental health is the development of computer-driven platforms that provide preliminary therapy through features such as self-assessment tools, virtual counseling, and stress management techniques [29]. In this way, people can communicate in various ways (via text, in-person, etc.), allowing the system to gather the necessary data to conduct the analysis. These AI tools direct students to professional resources, such as psychiatrists or helplines, ensuring timely intervention and providing comprehensive care.

9. Internship Management

Portfolio analysis: AI can be implemented in evaluating and analyzing internship portfolios by exploring a broad range of performance metrics such as task completions, reflection writing, and supervisor evaluations [11]. AI algorithms can learn patterns and identify outliers, offering interns the best feedback by utilizing the results in areas such as clinical competencies, professionalism, and communication skills. They can also provide complete analyses of these systems, which will improve education quality by guiding students to engage in effective career development.

Internship tracking: AI can track students’ progress in internships through data collection and analysis from various touchpoints such as daily logs, supervisor evaluations, and task completion rates [30]. AI systems deliver real-time insights by instantly identifying students in situations in which better practices are needed and suggesting a personalized set of strategies for their development. Complete AI-generated reports serve as powerful tools for academic records and help institutions identify the outcome of their internship programs, collaborate, and devise improved plans for future cohorts.

Placement optimization: AI-based platforms can be used for internship placement; these systems will enroll students with standard qualifications by analyzing the combination of their skills, academic performance, and career aspirations [31]. Using technological infrastructure, they manage and track data from students who have completed extracurricular activity assessments, aligning their preferences with final pairings. Moreover, entering students would receive daily guidance on their work, and these tools will ensure equal access to opportunities across institutions.

10. Student Activities

AI-driven clubs: AI-related student clubs can inspire and promote AI technologies by encouraging students to imagine, experiment, and implement AI-driven solutions [32]. These clubs can also provide the student body with a list of A-level coursework to give them experience in handling tools and methodologies. Furthermore, students can brainstorm local AI projects, including IoT-related topics, robotics involving drones, and software simulating town-wide energy storage. They may also have access to AI-controlled robots through the school’s maker space.

Competitions and hackathons: AI has allowed significant scientific and technological progress, encouraging open challenges addressing AI-related problems in health and education [33]. These events would allow students to practice their teamwork skills in real-life situations using AI tools and methodologies. They will also concentrate on other aspects, such as predictive analytics, AI-powered diagnostic tools, and virtual patient management. In addition, these competitions will bring together students from various fields, such as medicine, engineering, and data science, to develop innovative IT solutions for the healthcare industry.

Discussion

Recommendations for AI Implementation

1. Educational Practical Applications

A. Developing case-based learning modules that are based on ethical and professional dilemmas and not only use real-world scenarios that reflect problems encountered by medical professionals but also simulate them [3]. AI-powered tools can also enhance these modules to analyze student responses, provide feedback on decision-making processes, and propose alternative solutions. For instance, AI can be used to present scenarios related to patient consent issues, dealing with medical errors, or facing family communication problems. These scenarios ensure that students develop a good understanding of professional ethics.

B. Creating collaboration scenarios in which students from different majors, such as medicine, nursing, pharmacy, and other healthcare disciplines, interact in a virtual domain. These semi-authentic simulations may contain teamwork scenarios likely to occur in the real world such as patient care conferences and emergency response situations. These events will foster collaboration and communication skills between different teams [4]. AI-driven platforms are smart enough to analyze group dynamics and provide participation records and decision-making feedback. The students will acquire the skills needed for cross-discipline collaboration.

C. Integrating interactive guides (IGs) on new topics of telemedicine, AI ethics, and algorithm transparency in tutorials [7]. These IGs will become more advanced by incorporating adaptive learning platforms and providing adaptive content and case scenarios to guide students through complex ethical challenges and technological applications in the medical field. Telemedicine guides may consist of in-virtue consultations, laws of data privacy, and remote diagnostic tools, ensuring that students fully understand modern medical practice.

2. Training Programs

Teachers can conduct workshops (e.g., collaborative project simulation) in which participants and students apply their knowledge of tools and reflect on their practical implications in education and work environments [34]. During these workshops, the participants must receive training in various AI tools, including adaptive platforms and diagnostic simulators related to data analytics software. Students can also view case studies and be involved in role-playing activities. Ideally, this will allow the participants to observe real-life AI applications in medical education. By examining obstacles, these meetings will enable the participants to master AI technologies effectively and incorporate them into their teaching and learning.

3. Creating a Comprehensive Library

Engaging modules can be developed by telling authentic stories and using case-based learning, allowing students to better understand these moral issues [35]. They will center on essential topics such as data privacy, algorithmic bias, and the responsible implementation of AI in clinical decision-making. Besides quizzes, discussions, and role-plays, the learners can actively participate in activities. Modules can also be improved through regular updates that convey the new ethical treatment guidelines and technological advances.

4. Ethical and Regulatory Frameworks

A comprehensive guide to data privacy and privacy-protecting, bias-free AI is needed to ensure the proper use of AI in medical school while respecting privacy [36,37]. Institutions should always include the newest AI governance and compliance practices in the guidelines to ensure that they align with the law and ethical standards. The guidelines should include methods for securing student and patient information, reducing biases in machine learning by improving algorithm transparency, and auditing AI systems to ensure fairness and accountability. These actions will help schools build trust with students and the community.

5. Scalability and Accessibility

The infrastructure system must support the needed capacity and output through cloud-based solutions and robust networks to cope with users’ growing demand [38]. Furthermore, institutions should focus on building highly flexible platforms that address different technological needs; these platforms should be accessible on various devices and accessible in remote and underserved areas. Focusing on two main goals - security and redundancy - is a logical and safe path forward.

6. Continuous Evaluation

AI systems should be provided with feedback from users, including evaluations of their functionality, to allow enhancement. Such feedback will include input from students, educators, and administrators to comprehensively assess performance. Institutions can employ analytic models and feedback from end users to identify weaknesses, modify the features to cope with evolving needs, and ensure that AI aligns with pedagogical objectives.

Limitations and future research

As a narrative review, our study does not follow a systematic methodology for literature selection, which may introduce selection bias. Additionally, while we aimed to provide a broad and updated perspective on GAI applications in medical education, the rapidly evolving nature of this field means that new developments may emerge beyond the scope of this review. Future research, including systematic reviews and empirical studies, will be essential to further validate and expand on these findings.

## Conclusions

Generative AI has increasing potential to transform medical education by enhancing personalization, efficiency, and innovation. Through advanced algorithms and machine learning, GAI can adapt educational content to individual learning needs, optimize administrative processes, and provide immersive training experiences. However, ethical challenges, such as data privacy, algorithmic biases, and equitable access must be addressed. Collaborative efforts between educators, policymakers, and technology developers will ensure that AI is integrated responsibly and effectively, providing future healthcare professionals with dynamic, fair education and training.

## References

[1] Strielkowski W, Grebennikova V, Lisovskiy A, Rakhimova G, Vasileva T (2024). AI-driven adaptive learning for sustainable educational transformation. Sustain Dev.

[2] Hamilton A (2024). Artificial intelligence and healthcare simulation: the shifting landscape of medical education. Cureus.

[3] Zheng B, Mavis B Linking theory to practice: case-based learning in health professions education. Designing Technology-Mediated Case Learning in Higher Education.

[4] Maluleke T, Benecke R, Oladejo S (2024). Cross-disciplinary mathematical modelling to benefit healthcare - could clinical pharmacology play an enabling role?. Br J Clin Pharmacol.

[5] Mittal U, Sai S, Chamola V, and Sangwan D (2024). A comprehensive review on generative AI for education. IEEE Access.

[6] Temsah O, Khan SA, Chaiah Y (2023). Overview of early ChatGPT’s presence in medical literature: insights from a hybrid literature review by ChatGPT and human experts. Cureus.

[7] Sharma S, Rawal R, Shah D (2023). Addressing the challenges of AI-based telemedicine: best practices and lessons learned. J Educ Health Promot.

[8] Masters K, Benjamin J, Agrawal A, MacNeill H, Pillow MT, Mehta N (2024). Twelve tips on creating and using custom GPTs to enhance health professions education. Med Teach.

[9] Iweuno BN, Orekha P, Ojediran O, Imohimi E, Tobias H (2024). Leveraging artificial intelligence for an inclusive and diversified curriculum. World J Adv Res Rev.

[10] Shoaib M, Sayed N, Singh J, Shafi J, Khan S, Ali F (2024). AI student success predictor: Enhancing personalized learning in campus management systems. Comput Human Behav.

[11] Tolentino R, Baradaran A, Gore G, Pluye P, Abbasgholizadeh-Rahimi S (2024). Curriculum frameworks and educational programs in AI for medical students, residents, and practicing physicians: scoping review. JMIR Med Educ.

[12] Williams P (2023). AI, analytics and a new assessment model for universities. Educ Sci.

[13] Sajja R, Sermet Y, Cikmaz M, Cwiertny D, Demir I (2024). Artificial intelligence-enabled intelligent assistant for personalized and adaptive learning in higher education. Information.

[14] Zheng K, Shen Z, Chen Z, Che C, Zhu H (2024). Application of AI-empowered scenario-based simulation teaching mode in cardiovascular disease education. BMC Med Educ.

[15] Onesi-Ozigagun O, Ololade YJ, Eyo-Udo NL, Ogundipe DO (2024). Revolutionizing education through AI: a comprehensive review of enhancing learning experiences. Int J Appl Res Soc Sci.

[16] Al Shuraiqi S, Aal Abdulsalam A, Masters K, Zidoum H, AlZaabi A (2024). Automatic generation of medical case-based multiple-choice questions (MCQs): a review of methodologies, applications, evaluation, and future directions. Big Data Cogn Comput.

[17] Jani KH, Jones KA, Jones GW, Amiel J, Barron B, Elhadad N (2020). Machine learning to extract communication and history-taking skills in OSCE transcripts. Med Educ.

[18] Singh S, Kumar R, Maharshi V, Singh PK, Kumari V, Tiwari M, Harsha D (2024). Harnessing artificial intelligence for advancing medical manuscript composition: applications and ethical considerations. Cureus.

[19] Hasani N, Farhadi F, Morris MA (2022). Artificial intelligence in medical imaging and its impact on the rare disease community: threats, challenges, and opportunities. PET Clin.

[20] Javvaji CK, Reddy H, Vagha JD, Taksande A, Kommareddy A, Reddy NS (2024). Immersive innovations: exploring the diverse applications of virtual reality (VR) in healthcare. Cureus.

[21] Najjar R (2023). Redefining radiology: a review of artificial intelligence integration in medical imaging. Diagnostics (Basel).

[22] Davuluri Davuluri, M M (2021). AI in education: personalized learning pathways using machine learning algorithms. International Meridian Journal.

[23] George B, Wooden O (2023). Managing the strategic transformation of higher education through artificial intelligence. Adm Sci.

[24] Wagner G, Lukyanenko R, Paré G (2022). Artificial intelligence and the conduct of literature reviews. J Inf Technol.

[25] Deekshith A (2020). AI-enhanced data science: techniques for improved data visualization and interpretation. International Journal of Creative Research in Computer Technology and Design.

[26] Bahroun Z, Anane C, Ahmed V, Zacca A (2023). Transforming education: a comprehensive review of generative artificial intelligence in educational settings through bibliometric and content analysis. Sustainability.

[27] Al-Jaf K, Öz C, Mahmud H, Rashid TA (2024). Leveraging chatbots for effective educational administration: a systematic review [Preprints]. Preprints.

[28] Ayeni OO, Hamad NMA, Chisom ON, Osawaru B, Adewusi OE (2024). AI in education: a review of personalized learning and educational technology. GSC Adv Res Rev.

[29] Wimbarti S, Kairupan BH, Tallei TE (2024). Critical review of self-diagnosis of mental health conditions using artificial intelligence. Int J Ment Health Nurs.

[30] Chen Y, Jensen S, Albert LJ, Gupta S, Lee T (2023). Artificial intelligence (AI) student assistants in the classroom: designing chatbots to support student success. Inf Syst Front.

[31] Herath D, Dinuwan C, Ihalagedara C, Ambegoda T (2024). Enhancing educational outcomes through AI-powered learning strategy recommendation system. Int J Adv Comput Sci App.

[32] Zhang K, Aslan AB (2021). AI technologies for education: recent research & future directions. Comput Educ Artif Intell.

[33] Rasool S, Ali M, Shahroz HM, Hussain HK, Gill AY (2024). Innovations in AI-powered healthcare: transforming cancer treatment with innovative methods. Bullet.

[34] Ng DTK, Leung JKL, Chu SKW, Qiao MS (2021). Conceptualizing AI literacy: an exploratory review. Comput Educ Artif Intell.

[35] Mouta A, Torrecilla-Sánchez EM, Pinto-Llorente AM (2024). Design of a future scenarios toolkit for an ethical implementation of artificial intelligence in education. Educ Inf Technol.

[36] Weidener L, Fischer M (2024). Proposing a principle-based approach for teaching AI ethics in medical education. JMIR Med Educ.

[37] Temsah A, Alhasan K, Altamimi I, Jamal A, Al-Eyadhy A, Malki KH, Temsah MH (2025). DeepSeek in healthcare: revealing opportunities and steering challenges of a new open-source artificial intelligence frontier. Cureus.

[38] Lopez Garcia A, De Lucas JM, Antonacci M, Castell WZ, David M, Hardt M (2020). A cloud-based framework for machine learning workloads and applications. IEEE Access.

